# Psychosocial support and HIV-related stigma intervention needs of adolescents living with HIV in southern Ethiopia: insights from adolescents, caregivers, and healthcare providers

**DOI:** 10.1080/16549716.2025.2576956

**Published:** 2025-11-05

**Authors:** Melkamu Merid Mengesha, Degu Jerene, Charlotte Castor, Cecilia Follin

**Affiliations:** a Department of Health Sciences, Faculty of Medicine, Lund University, Lund, Sweden; b School of Public Health, College of Medicine and Health Sciences, Arba Minch University, Arba Minch, Ethiopia; c KNCV Tuberculosis Foundation, Tuberculosis Elimination and Health System Innovation, The Hague, The Netherlands; d Department of Oncology, Skåne University Hospital, Lund, Sweden

**Keywords:** formative qualitative study, digital peer support, exchange experience with peers, HIV status nondisclosure, mental health

## Abstract

**Background:**

HIV-related stigma interventions targeting adolescents living with HIV (ALHIV) are limited. When available, most of these interventions operate at a single socioecological level and target a single stigma domain, and participatory design is rare.

**Objective:**

This study aimed to explore the psychosocial support intervention needs of ALHIV from the perspectives of adolescents, caregivers, and healthcare providers, and to develop a digital anti-HIV-related stigma intervention.

**Methods:**

A formative qualitative design with 27 in-depth interviews was conducted in three hospitals that provide an in-clinic psychosocial support to ALHIV in southern Ethiopia. Audio-recorded interviews were transcribed verbatim, translated, and analysed using NVivo 14.0. Codes emerged from the data, leading to the development of categories and subcategories through qualitative content analysis.

**Results:**

The results are presented in three categories: coping with today, lowering barriers through knowledge and education, and worrying about future relationship. Fear of rejection and lack of trust led ALHIV to keep their status secret. Although adolescents were optimistic about their future in areas like education, achieving goals, and employment, they worried about forming relationships. Peer support groups offered valuable experiential support but struggled with geographical and logistical barriers. Digital tools emerged as a promising solution to overcome these barriers and enhance peer support group networking.

**Conclusion:**

Addressing nondisclosure reasons, such as fear of rejection and lack of emotional preparedness, and enhancing supportive networks can empower adolescents and strengthen their resilience. Their willingness to disclose and educate others can lay the groundwork for creating a stigma-free environment.

## Paper context

● **Main findings**: Adolescents living with HIV refrain from disclosing their HIV status beyond close circles for fear of rejection and stigma, which calls for their empowerment to cope with daily challenges and build resilience.

● **Added knowledge**: Adolescents’ recognition that the community stigmatises because of a lack of knowledge, combined with their motivation to teach others, can be leveraged to empower them and foster a stigma-free environment.

● **Global health impact for policy and action**: As stigma significantly affects the HIV continuum of care, findings in the current study will inform future anti-HIV stigma interventions.

## Background

Globally, about 2.58 million children and adolescents (<19 years old) are living with HIV, of whom 1.65 million are adolescents aged 10–19 years [1,2]. Eighty-five percent of the HIV cases among children and adolescents worldwide live in sub-Saharan Africa [2]. In Ethiopia, around 603,500 people are living with HIV across all age groups, including 33,000 children aged 0–14 years, with a significant variation across regions [3].

There are global ambitions to achieve the three 95 s – awareness, treatment and viral suppression targets for HIV – by 2030, as outlined in the 95-95-95 target [1]. However, children are falling behind adults in progress towards these targets, with rates 63-57-43 compared to 86-76-71 for adults [1]. HIV-related stigma is considered one of the key barriers in this disparity [4,5]. Based on Goffman’s work [6], Link and Phelan described stigma as a social phenomenon that involves the co-occurrence of processes of labelling differences, stereotyping such differences, separation, and discrimination vis-à-vis the exercise of power [7–9]. Stigma against adolescents living with HIV (ALHIV) has been a significant challenge in schools, with reports of name calling and the use of discriminatory language [10,11], at home, through separating utensils and sending adolescents to inferior schools [11–13], and in healthcare settings through disrespect [14]. Stigma and disclosure concerns were highlighted in a recent review as the most reported psychosocial challenges among ALHIV [5].

Stigma and discrimination due to HIV status may result in a poor engagement in care and medication adherence among adolescents. There is also a risk of developing a negative self-image, and becoming more concerned about the future [10,11,15]. To avoid such negative outcomes and to maintain acceptance by peers [16–18], adolescents try to keep their HIV status secret [10,11,13]. However, studies have indicated an association between poor coping strategies and negative mental health outcomes, including diminished psychological well-being [11,19]. Providing adolescents with sources of resilience and support is crucial [20], as they are in a vulnerable stage and often lack self-efficacy, coping skills, and social power [21].

In-clinic-based psychosocial support (PSS) platforms provide adolescents with opportunities to build relationships, stay engaged in health care, improve clinical outcomes, gain knowledge, reduce isolation and feelings of sadness, and develop coping strategies [13,14,16,22]. However, these platforms lack geographical inclusivity and fail to accommodate those who avoid in-person sessions due to fear of being seen by others. The platforms face logistical challenges and frequent interruptions, and often require adolescents to travel long distances [23,24]. A digital peer support intervention could address these barriers by enhancing accessibility, preserving anonymity, reducing travel costs, and increasing convenience for users [25].

The World Health Organization recommends integrating PSS interventions into existing HIV services and implementing them through various delivery approaches. These include the use of digital platforms to reach adolescents and young people where they are located [26]. However, integrated in-clinic programmes often lack accessible or expanded communication platforms to effectively reach adolescents across geographical boundaries. Existing HIV-related stigma research interventions targeting children and ALHIV typically focus on a single socioecological level, lack a participatory design, target only one single stigma domain, and are often not integrated into existing service delivery structure [4,27]. Participatory design entails the involvement of stakeholders (i.e. patients, caregivers, healthcare providers) at each phase of the research process so that their needs and expectations are expressed, to shape the strategy and outcomes of research in ways that meaningfully impact practice [28,29].

This study explored the psychosocial and HIV-related stigma intervention needs of ALHIV, as perceived by adolescents, caregivers, and healthcare providers (HCPs) in southern Ethiopia. The planned intervention, informed by findings from this study, will be integrated into existing HIV services to address self-and anticipated-HIV-related stigma. The intervention will involve ALHIV who were previously unable to participate due to anonymity concerns or geographical distance from the in-clinic PSS programme centre. Additionally, the digital platform will connect in-clinic participants across different locations.

## Methods

### Setting

We conducted this study in three hospitals in southern Ethiopia that provide in-clinic psychosocial peer support group programmes to children and ALHIV. The existing PSS programme is offered to adolescents who are aware of their HIV status. Adolescent champions, with a strong record of clinical outcomes in terms of CD4 count and viral suppression, received training and served as role models by leading the PSS sessions. These sessions were based on modules from the national guidelines developed by the Ministry of Health and its partners. HCPs trained in adolescent HIV care and PSS conduct clinical assessments and facilitate discussion sessions.

### Design

A formative qualitative design [30] was used to explore the psychosocial and HIV-related stigma intervention needs of ALHIV from the perspectives of adolescents, caregivers, and HCPs in southern Ethiopia. The study adheres to the Consolidated Criteria for Reporting Qualitative Research (COREQ) [31] (Supplementary File 3).

### Sampling and recruitment

Purposive sampling was used to recruit 27 study participants from three hospitals, with six adolescents, two caregivers, and one HCP from each study site (18 adolescents: 8 girls and 10 boys; six caregivers of adolescents: five females and one male; three HCP responsible for PSS and HIV services: one female and two males). The adolescents included in this study were aged 14–19 years, aware of their HIV status, and were taking antiretroviral medication. The HCPs recruited participants based on the above eligibility criteria and then introduced the recruited participants to the data collector (MM, male) for consent and interview. None of the recruited participants refused to take part in interviews. All participants, except the HCPs, received a transport fee at the end of each interview as reimbursement.

### Data collection

The in-depth one-on-one interviews were conducted with 27 participants using a digital audio recorder in private consultation rooms within the hospitals from 21 October 2023, to 4 November 2023. On average, the interviews lasted for 28–35 minutes. We believe the study sample size is adequate to achieve saturation based on Hennink et al.’s recommendations [32]. Hennink et al. suggest at least nine in-depth interviews for code saturation (i.e. no new issues are raised in the data) and 16–24 for meaning saturation (i.e. whether available data is sufficient to fully understand the issues raised).

The first author (MM, male), who holds a Master of Public Health degree and has experience with qualitative methods, conducted all the interviews and took fieldnotes. The data collector participated in the study solely as a researcher and had no professional or caregiving relationship with any of the participants. Separate interview guides were used for interviews with adolescents, caregivers, and HCPs. A pilot test of the interview guides was conducted before the actual interviews, to test for possible correction and ease of understanding. The interview guides together with the sociodemographic sections are provided as a supplementary file (Supplementary File 1).

### Analysis

Verbatim transcriptions into Amharic and the corresponding translations into English were conducted by the first author (11/27 interviews) and research assistants (16/27 interviews) with experience in qualitative data transcription and translation. Two authors (MM and DG) checked the consistency of the audio-recorded interviews, the verbatim transcriptions, and the English-translated versions of all the interviews. An inductive qualitative content analysis was applied, following the three phases of the inductive content analysis approach as described by Elo and Kyngäs, allowing categories and subcategories to flow from the data [33]. All the authors read the translated English versions to familiarise themselves with the data. Two authors (MM and CF) independently conducted the initial open coding and then discussed similarities and differences to produce a list of agreed-upon codes. The coding process included identifying meaning units, creating condensed meaning units that maintained participants’ original terms, and then assigning words or phrases that the authors believed represented the text in the condensed meaning unit (Supplementary File 2). The subsequent analysis was based on the final codes agreed upon by all the authors. Codes were thereafter grouped and abstracted by the creation of generic, and finally, main categories. The categories were discussed and agreed upon by all the authors. NVivo 14 was used to facilitate coding and categorisation.

### Reflexivity

When assessing the trustworthiness of qualitative studies [33], it is essential to consider the authors’ preconceptions. To minimise potential biases, coauthors from diverse clinical backgrounds in chronic health conditions among children, particularly HIV, and with qualitative research experience were included, and consensus was sought at multiple stages of the analysis. Finally, the author who conducted the interviews (MM, male) had neither a professional nor caregiving relationship with any of the study participants.

## Results

### Participant demographics

A total of 27 participants comprising 18 adolescents (eight girls and ten boys), six caregivers (four biological mothers, one grandmother, and one biological father), three HCPs (one female and two males) participated in in-depth interviews. The mean age of the adolescents was 17.1 years, only 33.3% lived with both parents, and 60.1% had a secondary education. In terms of participation in the in-clinic PSS programmes, 17% did not participate (Table 1).

**Table 1.** Participant demographics.

Characteristics		n (%)
Interviewees, *N* = 27	Adolescent living with HIV	18 (66.7)
	Caregivers	6 (22.2)
	Healthcare providers in ART clinics	3 (11.1)
Age in years	Adolescents, mean (SD)	17.1 (±1.7)
	Caregivers, mean (SD)	42.2 (±9.8)
	Healthcare providers, mean (SD)	34.3 (±8.7)
Adolescent currently living with, *N* = 18	Both parents	6 (33.3)
	Mother alone	8 (44.4)
	Grandmother	3 (16.7)
	Brother	1 (0.6)
Adolescent participation in peer support group, *N* = 18	Yes	15 (83.3)
	No	3 (16.7)
Adolescent education (grades attended), *N* = 18	5–8	7 (38.9)
	9–10	7 (38.9)
	11–12	4 (22.2)
Caregiver educational status, *N* = 6	No formal education	1
	Primary (1–8)	4
	Secondary (9–12)	1
Residence of adolescents	Urban	16 (89%)
	Rural	2 (11%)

SD = standard deviation; ART = antiretroviral therapy.

### Main categories and subcategories

The needs of ALHIV are described in three main categories: coping with today, lowering barriers through knowledge and education, and worrying about future relationship. The categories and subcategories are presented in Table 2. The categories are based on 201 relevant reference quotes from respondents, 100 from male and 101 from female respondents.

**Table 2.** Categories and subcategories of psychosocial support and HIV-related stigma intervention needs among adolescents living with HIV.

Category	Subcategory
Coping with today	Strive to keep HIV status secret
	Face mental health challenges
	Meet negative attitudes and discrimination from society
Lowering barriers through knowledge and education	Exchange experience with peers
	Welcome access to digital tool
	Seek for broader support
	Change perspective based on knowledge
Worrying about future relationship	Have hope despite concerns about the future
	View supportive family and clinic environments as instrumental

### Coping with today

Adolescents emphasised the need to keep their HIV status a secret, with reservations about disclosing it during emergencies or to someone they deeply trust. Life stressors stem not only from living with HIV but are also related to family conditions and negative societal attitudes, leading to self-stigma and anticipated stigma.

#### Strive to keep HIV status secret

Disclosure of HIV status was experienced as a struggle, with a feeling of insecurity about who to trust with sensitive personal information. There is broad consensus that disclosure of HIV status should not go beyond the family level. Lack of information control, fear of rejection and stigma, lack of emotional preparedness, and lack of confidence and skills to handle disclosure were expressed as barriers to self-disclosure. While some adolescents had the intention to disclose, others did not see or support the idea of the need to disclose and preferred to save it for emergency situations. Both boys and girls questioned the need to share HIV status information and argued that the disadvantages outweigh the advantages. Despite healthcare providers believing that disclosure benefits not only the individual but also others, they cautioned adolescents not to disclose until they were mature enough. They also suggested adolescents to be prepared for potential negative reactions following disclosure.

Disclosure should not go beyond the family. If it’s heard outside the family, the information will be spread, and children will reject friendships and walk away. *(Female adolescent, 15 years)*

I do not intend to disclose to others. I would rather take care of it personally. I will benefit nothing from telling others; I could add or reduce nothing for others if I tell them. *(Male adolescent, 19 years)*

I believe the disadvantage of disclosure outweighs the advantage. Until now, when people find out you have HIV, they tend to reject you. So, you must keep it a secret. Trust me, there is no advantage. (Female adolescent, 19 years)

#### Face mental health challenges

When adolescents were informed of their HIV diagnosis at an older age, they reported that they had been treated dishonestly about the reasons for their medication and struggled to cope with learning they were infected. Furthermore, strong negative thoughts about the meaning of life and the impact of lifelong medication were highlighted. The perception of being different and inferior due to living with HIV varied among boys and girls: some had previously felt this way, some still experienced it, while others had never felt it. Adolescents expressed their emotional reaction to what others say about the condition they are living with, as in ‘when some people hear about this disease (HIV/AIDS), they make you feel ashamed *[the respondent breaks down in tears]*’ (Female adolescent, 16 years).

Sometimes, I feel isolated and different when the family speaks at me; I think they do so because I’m sick and different. Sometimes, when I’m alone and deep in thought, I break into tears and just feel unhappy. I have a feeling that I’m different from others. *(Female adolescent, 14 years)*

I used to consider myself inferior to others due to my HIV status and perceived that my friends may discriminate, so I didn’t meet them. I can now, however, do things beyond my friends and learn like they do. *(Male adolescent, 19 years)*

I’ve never judged myself because of HIV and don’t feel inferior because I’m living with HIV … I’ve been living with HIV since my childhood, and I don’t feel anything different. *(Female adolescent, 19 years)*

Adolescents reported feelings of alienation and unhappiness, which were evident even within their families. Some also carried the additional burden of supporting themselves and younger siblings due to a dysfunctional family, which led to feelings of hopelessness and questioning why they must endure such hardships. The coping strategies adolescents employed to manage their emotions included meeting friends, participating in peer support groups where they could discuss HIV without judgment, seeking advice from healthcare professionals, and turning to spirituality.

#### Meet negative attitudes and discrimination from society

Stigma within the community, school, and home was highlighted as a pervasive issue, leaving adolescents with a sense of insecurity. The adolescents stressed the lack of acceptance in society and how their life was affected by people’s attitudes. Caregivers observed a decline in negative attitudes toward individuals living with HIV. Meanwhile, both HCPs and adolescents noted that many still perceive individuals living with HIV as unclean and living in a hopeless condition, although these negative attitudes vary from person to person within the community.

If a person reveals their HIV status, people take it very seriously and exaggerate it. They fear eating or drinking with that person. It’s this that holds back adolescents from sharing their HIV status information with others. *(Female healthcare provider, 44 years)*

If they know that I’m living with the virus, they may distance from me and speak bad words at me. Once they learn that I live with the virus, they could never approach me. *(Female adolescent, 16 years)*

Some people judge you as having HIV if you are thin by merely looking at you. If you’re fat and well dressed, they assume that you don’t have it. Life is difficult like people’s perception. In terms of HIV, they (other people) are not ready to accept it, that is it. *(Female adolescent, 19 years)*

At home, negative attitudes are expressed through various forms of discrimination, such as separating utensils and beds, despite these items posing no risk of transmission. Additionally, adolescents faced name-calling in schools during disagreements with friends.

… for example, a female adolescent came to me and was crying a lot … when she quarrelled with a friend in school … her friend insulted her using the virus, saying that she had HIV. *(Female healthcare provider, 44 years)*

### Lowering barriers through knowledge and education

Respondents discussed extensively about the importance of exchanging experiences with peers as a source of confidence, knowledge, and responsibility for improved medication adherence and self-care. The digital platform was recognized for its potential to deliver health information and host peer support group discussions; however, gaps in digital literacy, access to digital tools, and privacy issues were emphasised as key concerns.

#### Exchange experience with peers

Peer support group discussions were described as an important source of encouragement, friendship, and HIV-related knowledge. These groups provided adolescents the freedom to openly discuss their condition, which in turn improved treatment adherence, strengthened personal responsibility, and fostered the belief that HIV is manageable like any other chronic condition. This shift in perspective enhanced their confidence in their ability to live a normal life.

My participation here (in the peer support group) improved my self-confidence, and I became aware that I can learn and work just like anybody else. *(Male adolescent, 18 years)*

Caregivers and healthcare providers emphasized that peer support groups should overcome geographical barriers and inequities in access, enabling adolescents to share their experiences and knowledge that foster a more positive life. At the same time, a caregiver reflected on the difficulty of persuading her urban-based son to participate, contrasting with the reality of rural adolescents who are eager to join but unable due to financial and geographical constraints.

ALHIV in different places might somehow have different experiences, so it could be good if they come here (hospital) to teach and share their experiences, and the ones here go to other places to share their experiences. *(Grandmother, 56 years)*

I often tried to get him [her son] to join and participate [in peer support group]. However, he never wanted to be part of such group … I’d be very happy if all children living with HIV came here (to the hospital) and exchanged thoughts and relieved their stress and felt free. *(Mother, 41 years)*

Adolescents from rural areas are disadvantaged because they do not receive this service (peer support). If they had joined this service, they would’ve had role models to relate to. Now that I’m leaving them behind, it has been an enormous challenge for me and for the service. *(Female healthcare provider, 44 years)*

#### Welcome access to digital tools

Adolescents discussed accessing health information from various sources, including digital platforms, but expressed a preference for HCPs as their main source for HIV-related information. HCPs, however, highlighted the potential of digital platforms to reach adolescents who might otherwise be excluded from peer support sessions.

I think such a digital platform could enable adolescents who are unable to physically attend PSS sessions. *(Male healthcare provider, 32 years)*

While HCPs trust the potential of digital platforms to deliver health information, they emphasised on the importance of agreeing on the message content and informing caregivers in advance. The HCPs also raised concerns related to information security of digital platforms, digital literacy, mobile phone access and family takeover issue in discordant families, and an increase in adolescents’ screen time.

A digital platform could enable adolescents who are unable to physically attend PSS sessions. It’s important, however, to ensure that they (adolescents) have a mobile phone, are educated, and can read texts they receive. (*Male healthcare provider, 32 years)*

Providing information via a mobile phone is fine. But the educational content sent over a mobile phone should be neutralised and a balanced one, without revealing our status. *(Male adolescent, 18 years)*

#### Seek for broader support

Adolescents expressed a strong need for more comprehensive information and practical skills that would restore hope and help them manage daily life with HIV. Caregivers supported this need, recommending that adolescents receive clear, expert-led explanations during clinic visits.

I need something that gives me strength … that’s all I need … that’s all I need. I simply need information that will help me regain hope. *(Female adolescent, 19 years)*

HCPs suggested that peer support programmes shift from a focus about forgetting HIV and feeling free to an approach that prioritises psychological care, problem-solving, and practical education to help adolescents cope effectively.

It should give priority to psychology … rather than only focusing on how they forget it [HIV] and feel free … it could be better if it helps them understand their condition and look for a solution. (*Male healthcare provider*, 27 years)

To sustain gains from peer support, healthcare providers discussed the significance of linking adolescents graduating from the PSS group to youth HIV associations where they can find more role models.

When adolescents graduate from the group, we link them with HIV associations. In the association, there are youths who revealed their HIV status and give lectures about HIV in review meetings. *(Female healthcare provider, 44 years)*

#### Change perspective based on knowledge

Initially, adolescents internalised society’s negative views on HIV. After receiving education through peer support groups, however, their understanding and ability to manage their condition improved. They described HIV as a manageable condition rather than a ‘killer disease.’ Yet they also recognized that broader societal acceptance remains limited, largely due to a lack of knowledge, leading them to prefer educating others before disclosure. They highlighted the range, from negative to positive, among people’s attitudes toward HIV, and highlighted their opinions that educated people are more receptive of people living with HIV.

Previously, since my childhood, I considered HIV as something horrible. However, after joining the peer support group, I understood that it is nothing and I can live like others do. (*Female adolescent, 18 years)*

Although there is no disease that is comforting, I consider HIV/AIDS is not something serious compared to other chronic conditions … . Society lacks knowledge and understanding about it and discriminates against those living with the virus in a way that is difficult to explain. (*Male adolescent, 16 years)*

Adolescents were often emotional when reflecting on how society treated people living with HIV. They described this mistreatment as rooted in fear rather than fact.

It’s very disgusting when a person is stigmatised for having a disease like this [HIV/AIDS]. People should offer help rather than marginalise. It’s terrible how they treat these individuals, and they should not be seen as evil. This disease is not as bad as they think. They’re afraid of the so-called “killer disease” discussed in lectures. *(Female adolescent, 16 years)*

### Worrying about future relationship

Adolescents did not consider HIV/AIDS as a ‘killer’ disease that limited them from achieving life goals. However, they were concerned about the limited options available when it comes to building relationships that lead to marriage.

#### Have hope despite concerns about the future

Adolescents emphasised that HIV does not prevent them from pursuing their life goals and expressed that they are ready to face challenges ahead. At the same time, they expressed deep concerns about the difficulties of disclosing their HIV status in future romantic relationships.

I don’t believe that this illness (HIV/AIDS) affects my ability to achieve my goals. However, it could affect sexual relationships and marriage. When I think of a sexual partner, most of them are HIV-negative and I worry about how to continue given my condition … . I then must end a relationship, and it hurts me in this regard. *(Female adolescent, 19 years old)*

Now I’m free, I value my antiretroviral drugs, commit to taking care of myself, ready to take my medicine and face whatever challenge in life, and I am ready. *(Male adolescent, 18 years)*

Assume that you can marry an HIV – negative woman and have a healthy baby. The question is, when you are at a friendship level, how can you engage in a talk that leads to marriage? That’s the influence on me. This is the only thing that gives me a heavy heart. May God help. *(Male adolescent, 16 years)*.

While most adolescents described worry about disclosure and acceptance, one participant offered a contrasting perspective, expressing confidence about future romantic relationship:

No, there’s nothing I’m concerned about regarding relationships. I will have a normal friend [not living with HIV], and if our relationship leads to marriage, we will bear children with caution and raise them in a good condition. *(Female adolescent, 14 years old)*

#### View supportive family and clinic environment as instrumental

Adolescents described family support as instrumental for emotional comfort, reminding them about medication and helping them maintain courage so they did not feel left behind. Caregivers emphasised the importance of encouraging adolescents to pursue their goals, safeguarding their mental health, and supporting them to become whatever they aspire to be.

The support of my father was particularly indispensable. He encouraged me to join this programme (the peer support group), provided me with emotional comfort, advised me not to miss appointments. (*Female adolescent, 18 years)*

If the biological parents are alive, they should, to the best of their ability, try to maintain the morale of adolescents to prevent them from becoming emotional about their condition. *(Father, 50 years old)*

A supportive clinical environment offered a close and regular contact for HIV/AIDS services and facilitated peer support discussions, addressing adolescents’ concerns and providing emotional support. HCPs expressed empathy with the adolescents’ struggles:

When they’re sad, we’re sad, and when they’re happy, we’re happy. We attempt to support them by following up, making phone calls, and connecting them to individuals and organisations that will support them. (*Male healthcare provider*, 27 years)

## Discussion

Perspectives of adolescents, caregivers, and health care professionals on PSS and HIV-related stigma intervention needs of ALHIV in southern Ethiopia are presented in three categories: *coping with today, lowering barriers through knowledge and education, and worrying about future relationship*. Stigma and discrimination are major challenges, leading to the nondisclosure of HIV status and impacting the mental well-being of adolescents. Broad support from health professionals, family, and peers is crucial for enhancing knowledge, which can boost confidence in discussing HIV and help shift societal attitudes toward individuals living with HIV. In this regard, participants recognised the potential of digital platforms, provided certain gaps are addressed. Furthermore, while adolescents expressed hope for the future – including pursuing education and having children – they also voiced concerns about finding a partner.

Lack of emotional preparedness, lack of confidence and skills to handle disclosure, fear of stigma and rejection, and lack of information control contributed to nondisclosure of HIV status beyond the family level. Previous studies have similarly reported fear of anticipated stigma as a reason for nondisclosure, despite adolescents’ perceived benefits of disclosure [34–36]. Lack of trust and fear of rejection due to stigma can lead to feelings of insecurity, negatively impacting adolescents’ mental well-being [37]. A study also highlighted that external HIV stigma can result in self-isolation, helplessness, school dropout, and avoidance of necessary healthcare [38]. Keeping the HIV status a secret and choosing nondisclosure, as reported in this study, has also been documented elsewhere to avoid negative consequences and maintain social acceptance [39]. However, such a maladaptive coping mechanism is associated with mental health symptoms, including stress and disclosure-related anxiety [19,37,40].

Consistent with our study findings, a systematic review reported stigma as the most common psychological challenge facing adolescents, particularly in school settings, from both their peers and teachers [5]. Similarly, our findings on the reasons behind stigmatisation align with existing evidence [11,38,41], indicating that stigma stems from fear of transmission, lack of knowledge, and misconceptions about HIV. This study underscores the need to empower adolescents to take the lead in fostering a stigma-free environment, aligning with their desire to shift societal attitudes toward people living with HIV. The call for empowerment is supported by the structuration theory, which posits that individuals have the power and agency to transform their circumstances [42].

Exchanging experiences with peers was the most discussed subcategory, with respondents emphasising that peer support enhanced knowledge, encouragement, emotional well-being, networking, and clinical outcomes. Similar findings have been reported in previous studies, highlighting the role of peer networks in fostering self-care and empowerment among ALHIV [16,34,35,43,44]. Suggested key psychosocial intervention strategies for effective PSS include addressing individual-level factors (e.g. agency, empowerment, and self-care skills), implementing tailored delivery methods (e.g. digital platforms), and providing structural resources [43]. In line with study participants’ expressed needs and the recommendations of Laurenzi et al. [43], our planned intervention will offer structural resources and digital literacy empowerment training to enhance the effective use of the Telegram mobile application. The Telegram mobile app will serve as a unified online platform, connecting peer support groups from across different health facilities to facilitate experience-sharing and knowledge exchange.

Addressing privacy concerns and delivery of a neutralised content that does not mention HIV was the top priority for adolescents accepting the intervention. This has also been reported previously in an eHealth intervention that aimed to promote medication adherence of ALHIV in the current study settings [45]. Consequently, to address the respondents’ concerns related to digital security, the built-in Telegram ‘privacy and security’ settings will be used, when developing the digital anti-stigma intervention and intervention contents will be deidentified of terms defining their condition. Moreover, the Telegram mobile app offers various chat input options, including text, voice, images, and audiovisual materials, providing adolescents with alternative ways to communicate if they struggle with one format, which narrows the digital literacy gap and improves engagement [46].

Following the recommendation of the World Health Organization [26] and researchers such as Laurenzi et al. [43] to integrate psychosocial interventions into existing care, and the use of digital platforms, our planned digital peer support-based intervention will operate from the existing healthcare system. We believe that the healthcare providers’ engagement, from identifying intervention needs to content validation, participant selection, and monitoring of adolescents’ participation in intervention sessions, could lay the foundation for future sustainable transition to ownership of the project.

This study is among a few that explored the psychosocial needs of ALHIV in Ethiopia within the context of peer support [35,36]. It provides valuable insights for developing interventions and optimising their delivery approaches. In line with the Medical Research Council framework recommendations [28], we explored the perspectives of various stakeholders. However, this study has certain limitations. Although we attempted to balance gender, age, and context-specific experiences, only a small number of rural adolescents and nonparticipants in PSS groups were included, which may limit the transferability of the findings. While our findings suggest that adolescents are facing additional life stressors beyond living with HIV, including family-related challenges, we did not examine how general living conditions influence their self-management and psychological adjustment. Therefore, the results should be interpreted with caution to avoid attributing their reported mental health challenges solely to HIV. Finally, conducting the interviews in a hospital setting may have influenced participants’ responses. However, this was a pragmatic approach, as conducting the interviews in their homes or other locations could have increased the risk of unintended stigmatisation, potentially discouraging participation.

## Conclusions

The findings underscore the importance of peer support groups in enhancing the clinical management of HIV/AIDS services and improving adolescents’ mental well-being. Addressing concerns related to HIV status disclosure and relationships, empowering adolescents to manage perceived negative societal attitudes toward people living with HIV, and providing tailored individual and social life skills training, could be crucial in meeting the PSS needs of ALHIV. Finally, addressing gaps in digital literacy, smartphone ownership, and privacy concerns could strengthen digital platforms as safe spaces for facilitating peer support group discussions and expand them beyond physical geographical limits.

## Supplementary Material

Supplementary file 2.docx

Supplementary file 3.pdf

Supplementary file 1.docx

## Data Availability

All data relevant to the study are included in the article or provided as supplementary information.

## Acronyms

AIDS, acquired immunodeficiency syndrome; ALHIV, adolescents living with HIV; ART, antiretroviral therapy; CD4, cluster of differentiation; COREQ, consolidated criteria for reporting qualitative research; HCP, healthcare provider; HIV, human immunodeficiency virus; SD, standard deviation.
