# Supplementary material for: Psychosocial support and HIV-related stigma intervention needs of adolescents living with HIV in southern Ethiopia: insights from adolescents, caregivers, and healthcare providers
Source: Glob Health Action. 2025 Nov 5;18(1):2576956. doi: 10.1080/16549716.2025.2576956 (PMC12590571; doi:10.1080/16549716.2025.2576956)
Supplement: Supplementary file 2.docx [file ZGHA_A_2576956_SM4977.docx]

Supplementary file 2: Supplementary file to illustrate the analysis procedures

| Selected relevant reference quotes | Condensed meaning unit | Code | Subtheme | Theme |
| --- | --- | --- | --- | --- |
| [<Files\\Interview texts\\AGH_Fadol_02>](file:///C:\Users\me1171me\Work%20Folders\Documents\Lund%20University\Manuscript%20from%20the%20PhD%20project\9174049c-d2cd-429f-addc-3bd984b144ce)  I didn’t tell [HIV status] anyone. But I take care of others. …. If I tell them, **they will not take it as a good thing that is why I don’t tell**. I want to tell but they are not good. I tried two or three times to tell them but since they are people of rumour I decided not to tell. | I didn’t tell others as people are not good and inclined to spread rumours, but I will take care for them. | Adjust life with friends without disclosing HIV | Keeping HIV status secret | Coping with today |
| [<Files\\Interview texts\\AGH_FHCP_01>](file:///C:\Users\me1171me\Work%20Folders\Documents\Lund%20University\Manuscript%20from%20the%20PhD%20project\9dda9550-a7c6-4e1a-b7dc-2a47e4d95105)  The other issue is, for your surprise, irrespective of their participation in the psychosocial group and whether that they become champions, none of them make self-disclosure that they have HIV in schools. Despite they come here for participation to get trained or as a champion, **most of them do not reveal to others that they are HIV infected**. Why is this? Because stigma and discrimination still exist in the community. | Most of the adolescents in the peer group including peer leaders didn’t make self-disclosure for fear of stigma and discrimination. | Non-disclosure | Keeping HIV status secret | Coping with today |
| [<Files\\Interview texts\\AGH_Madol_05>](file:///C:\Users\me1171me\Work%20Folders\Documents\Lund%20University\Manuscript%20from%20the%20PhD%20project\d62c355b-484d-4def-82db-f6765d967567)  I want to tell but I’m afraid. I can tell a person I believe that I trust, but how much does that person trusts me after learning my HIV status? | I want to tell my HIV status to someone whom I trust but I am afraid that how long that trust remains. | Lack of trust | Mental health challenges | Coping with today |
| [<Files\\Interview texts\\AGH_Madol_01>](file:///C:\Users\me1171me\Work%20Folders\Documents\Lund%20University\Manuscript%20from%20the%20PhD%20project\ca800d3c-20fc-40e6-a7dc-3d310707e5f1)  I **feel depressed when I am alone**. If I gather with other people, then I feel happier. I feel very happier when I chat with others. I may feel sad when depressed or no one around. | I feel happy when I am with other people or chat with them, otherwise, I feel depressed when alone. | Happy when not alone | Mental health challenges | Coping with today |
| [<Files\\Interview texts\\JGH_Fadol_02>](file:///C:\Users\me1171me\Work%20Folders\Documents\Lund%20University\Manuscript%20from%20the%20PhD%20project\79c08260-350d-4c64-a8dc-2a4852cfdb93)  As far as I’m physically well, irrespective of living with the virus. I can do whatever HIV free people can do. Nothing hinders me doing as far as my body is not injured or I’m disabled. | If I am physically well, irrespective of living with the virus, I can do whatever HIV free people can do. | Nothing holds me back | Self-confidence | Coping with today |
| [<Files\\Interview texts\\JGH_Madol_06>](file:///C:\Users\me1171me\Work%20Folders\Documents\Lund%20University\Manuscript%20from%20the%20PhD%20project\9b025851-6bcb-4f3a-b8dc-07f83fa63e11)  Living with HIV did not affect our capacity to overcome challenges. What has it affected us so far? If we do not take the medicine properly, there are many problems that can affect us. Many of them show signs, so if we take the medicine properly, **we can live like a healthy person** and even longer years. | If we fail to take medications appropriately, many problems can occur. But if we take them appropriately, healthy life with long years is possible. | Healthy life with appropriate medication  medications for success | Self-confidence | Coping with today |
| [<Files\\Interview texts\\JGH_Fadol_03>](file:///C:\Users\me1171me\Work%20Folders\Documents\Lund%20University\Manuscript%20from%20the%20PhD%20project\cb1c0512-e23e-42b8-9bdc-3d2f51ed653e)  **People still hold the belief that people living with HIV are inferior**. However, if a person living with HIV takes medication appropriately, there is no difference and hence equal. It would be good if people could move away from this misconception. **They still believe HIV is a killer**. | People still hold the belief that a person with HIV is inferior and consider HIV is as a killer. | Other people’s preconception of living with HIV | Negative attitude from the society | Coping with today |
| [<Files\\Interview texts\\JGH_Fadol_05>](file:///C:\Users\me1171me\Work%20Folders\Documents\Lund%20University\Manuscript%20from%20the%20PhD%20project\9746ae95-9d80-4b0f-b0dc-3d2f6cd13e5b)  Most of the time they think of it as a difficult disease, a dirty disease. Some people judge you as an HIV positive if you are thin merely by looking at you. If you are thin, they assume you have HIV; if you are fat and well-dressed, they assume you do not. **Life is difficult, like people's perception.** In terms of HIV, the **people are not ready to accept it**. That is, it! | People think HIV as a difficult and dirty disease and are not ready to accept. They judge you as having HIV if you look thin. | Society not accepting HIV | Negative attitude from the society | Coping with today |
| [<Files\\Interview texts\\AGH_Madol_01>](file:///C:\Users\me1171me\Work%20Folders\Documents\Lund%20University\Manuscript%20from%20the%20PhD%20project\ca800d3c-20fc-40e6-a7dc-3d310707e5f1)  After I came here, and joined the peer-support group, I have **gained a lot of knowledge and understanding** through sharing lived experiences of one another in the group. | I have gained a lot of knowledge and understanding in peer-support group through shared lived experiences. | Knowledge and understanding | Exchange experiences with peers | Lowering barriers through knowledge and education |
| [<Files\\Interview texts\\SGH_FCG_01>](file:///C:\Users\me1171me\Work%20Folders\Documents\Lund%20University\Manuscript%20from%20the%20PhD%20project\6a1565a1-cad2-4dba-b3dc-25d71adae70a)  From the government, what I think is that adolescents living with HIV **get experiences from different places**, in a form of training it could be, than be limited to a one place. Adolescents living with HIV in different places might somehow different experiences, and hence, it could be good they come here to teach and share their experiences, and the ones here go to other places to share their experiences. It is my wish that the government does this. | Adolescents should get experiences from different places than limited to experiences at one health facility. | The way forward | Exchange experiences with peers | Lowering barriers through knowledge and education |
| [<Files\\Interview texts\\AGH_FCG_02>](file:///C:\Users\me1171me\Work%20Folders\Documents\Lund%20University\Manuscript%20from%20the%20PhD%20project\4aef0b3e-1d49-4f4d-a2dc-2a47e4ae1011)  I would be very happy if all children living with HIV including those who are not able to come, and the poor, come here and **exchange thoughts and get relieved of stress** and feel free. | I appreciate adolescents living with HIV participate in the peer group and exchange thoughts and get relieved from stress. | Program acceptance | Exchange experience with peers | Lowering barriers through knowledge and education |
| [<Files\\Interview texts\\JGH_HCP_01>](file:///C:\Users\me1171me\Work%20Folders\Documents\Lund%20University\Manuscript%20from%20the%20PhD%20project\d11256bb-4fbe-483e-a0dc-3d2fc85e0150)  The children themselves come with different characteristics -- **they come with a lot of problems**; come with the questions faced at their neighbourhood, at school. We cannot address them all. | Children come from different backgrounds with different questions in their daily life and we cannot address them all. | Knowledge and needs | Need of support | Lowering barriers through knowledge and education |
| [<Files\\Interview texts\\JGH_Madol_01>](file:///C:\Users\me1171me\Work%20Folders\Documents\Lund%20University\Manuscript%20from%20the%20PhD%20project\46661de2-ff0a-4394-b5dc-2a4853084bf9)  There are many things we don't know. We would love it if they could **clarify things we didn't learned before**... explaining different things. | We would love to learn many things we don’t know before with explanations. | Knowledge and needs | Need of support | Lowering barriers through knowledge and education |
| [<Files\\Interview texts\\AGH_Madol_01>](file:///C:\Users\me1171me\Work%20Folders\Documents\Lund%20University\Manuscript%20from%20the%20PhD%20project\ca800d3c-20fc-40e6-a7dc-3d310707e5f1):  When I talked about my disease to HIV negative friends, they couldn’t believe that I have HIV and criticized why people take it seriously. Some in society think people living with HIV have something posted on their forehead. This is **due to lack of sufficient knowledge and** **discriminating against those living with the HIV in a way that is difficult to explain** | HIV negative friends don’t believe me when I tell them about my disease and criticize why people take it seriously. The discrimination, due to lack of sufficient knowledge, is difficult to explain. | Attitude from the society | Discriminating due to lack of knowledge | Lowering barriers through knowledge and education |
| [<Files\\Interview texts\\JGH_Madol_06>](file:///C:\Users\me1171me\Work%20Folders\Documents\Lund%20University\Manuscript%20from%20the%20PhD%20project\9b025851-6bcb-4f3a-b8dc-07f83fa63e11)  There are those who think good and there are still others who think bad. **Those who think good are the ones who are educated** and know about everything. **People who don't know anything about the medicine [ART] or the disease are the ones who offend**. | Compared to educated people, it is those who don’t have understanding about the disease who speak offending words. | Attitude from the society | Discriminating due to lack of knowledge | Lowering barriers through knowledge and education |
| [<Files\\Interview texts\\SGH_Madol_05>](file:///C:\Users\me1171me\Work%20Folders\Documents\Lund%20University\Manuscript%20from%20the%20PhD%20project\8daeab66-bc63-44f8-8ddc-46be872dd5e8)  I **primarily get health information from the healthcare providers** and discuss with them. If there are still something else that I need to know I use my cell phone to access information. I use my own phone to google the internet and access HIV related information for the training. However, I primarily prefer to seek health information from the healthcare workers. | I primarily get health information from healthcare providers, and I consult the internet for further information. | Primary source of information | Access to digital tool | Lowering barriers through knowledge and education |
| [<Files\\Interview texts\\SGH_HCP_01>](file:///C:\Users\me1171me\Work%20Folders\Documents\Lund%20University\Manuscript%20from%20the%20PhD%20project\17758212-1cc2-405b-b8dc-4e8f0b5990cd)  I think such a digital platform could be possible to let adolescents who are unable to physically attend the psychosocial support session. It is important, however, to **make sure that they have a mobile phone, they are educated, and able to read texts they receive**. Otherwise, there could be no big challenge in this regard. | Digital platforms could be helpful for adolescents who are not able to attend the in-person psychosocial support sessions, but they need to have a mobile phone and digital literacy. | Digital literacy | Access to digital tool | Lowering barriers through knowledge and education |
| [<Files\\Interview texts\\AGH_FCG_01>](file:///C:\Users\me1171me\Work%20Folders\Documents\Lund%20University\Manuscript%20from%20the%20PhD%20project\67e9b178-09cc-4af8-a5dc-2a47e47dd0ab)  I advise her to make her mind free and **do not worry what others could say**. It is important that she doesn't worry about her health condition while joining her friends in crowd. She is taking a single dose, and who knows the Living God will make this condition go away for all. | I advise her to free her mind and not to worry about her health while being with friends. | Emotional support | View supportive family and clinic environments as instrumental | Worrying about future relationship |
| [<Files\\Interview texts\\AGH_Fadol_02>](file:///C:\Users\me1171me\Work%20Folders\Documents\Lund%20University\Manuscript%20from%20the%20PhD%20project\9174049c-d2cd-429f-addc-3bd984b144ce)  **Being here encourages me** further. At the first day when I see peoples, I was scared. I said oh no, I was scared on the first day, but the (healthcare provider) said “don’t fear these all have the virus in their blood,” and then I decided not to fear any longer. | I was scared when joining the group for first time, but healthcare providers encouraged me. | Encouragement from healthcare providers  From fear to safe | View supportive family and clinic environments as instrumental | Worrying about future relationship |
| [<Files\\Interview texts\\SGH_Fadol_04>](file:///C:\Users\me1171me\Work%20Folders\Documents\Lund%20University\Manuscript%20from%20the%20PhD%20project\d2dd9552-462b-4ae7-a9dc-251a2a196d87)  There is nothing that worries me of living with HIV! Because there are **many role models who are like me** but get educated and attained their life goals. I can marry a person who is also living with HIV. I believe I will achieve a life of success and help my parents and I don’t have such negative feelings because of my HIV status. | There is nothing that worries me as there are several roles models who achieved success while living with the virus. | Inspiration from role models | Have hopeful despite concerns about the future | Worrying about future relationship |
| [<Files\\Interview texts\\SGH_Fadol_01>](file:///C:\Users\me1171me\Work%20Folders\Documents\Lund%20University\Manuscript%20from%20the%20PhD%20project\a2edc2b3-9cd2-401a-92dc-3d4c6148af35)  If I marry to a man, he must be also living with the virus; my grandmom advised me so. The newborn will be prevented from getting infected through medications. I know it is possible to have a healthy baby in a marriage with a person living with HIV. | Grandmom advised me to marry a person with HIV and I know that we can have a healthy baby. | Narrow future partners | Have hopeful despite concerns about the future | Worrying about future relationship |
