# Supplementary material for: Psychosocial support and HIV-related stigma intervention needs of adolescents living with HIV in southern Ethiopia: insights from adolescents, caregivers, and healthcare providers
Source: Glob Health Action. 2025 Nov 5;18(1):2576956. doi: 10.1080/16549716.2025.2576956 (PMC12590571; doi:10.1080/16549716.2025.2576956)
Supplement: Supplementary file 1.docx [file ZGHA_A_2576956_SM4975.docx]

***Socio-demographic and clinical characteristics of adolescents***

***Date of interview ________________Name of the hospital _____________________________***

| **#** | **Item** | **Response** |
| --- | --- | --- |
|  | **Background Characteristics** |  |
|  | Respondent gender | \| ___ \| |
|  | How old were you on your last birthday? **(Record age in complete years)** | \| ___ \| years |
|  | What is your current marital status? | 1. Never married. 2. Currently married. 3. Divorced/widowed. |
|  | Are you currently attending school? | 1. Yes 2. No |
|  | What school grade you have already completed? | \| ___ \| |
|  | Are your biological parents alive? | \|______________________\| |
|  | With whom are you currently living with? | \|______________________\| |
|  | **Clinical information** |  |
|  | Adolescent interview code (To be used to link with caregiver interview) |  |
|  | Do you know the name of the disease condition for which you are taking medication? (Confirm the child knows his/her status)? | 1. Yes 2. No🡺do not proceed |
|  | Could you share with me how you acquired HIV? Was it through birth or later in life? | 1. Through birth 2. Later in life |
|  | For how long you have been in the psychosocial group (in months) | ____________ |

***Qualitative study topic guides***

***KII Guides: Adolescents’ perspectives on psychosocial health support needs***

1. If you have attended, what is your experience of attending the in-clinic psychosocial support program? Can you describe the lessons you learned and the challenges you have experienced?

(Probe: explore reasons for non-attendance if s/he is not participating in the in-clinic psychosocial support group.)

1. What HIV-related health information needs have you got? Do you have some additional needs?
2. What is your experience of accessing health information about HIV from sources other than the in-clinic psychosocial support? Can you describe your experience of accessing such information using digital technology??

(Probe: discussion with others (including family member) regarding the health information received related to your condition)

1. What are your thoughts on the type of information and content that should be included in digital health platform for adolescents living with HIV? Please share your insights on what would be most beneficial for you in such platform.

(Probe: explore willingness to receive HIV-related health information through their mobile phone)

1. What are some of the facilitators and challenges do you encounter when accessing and utilizing digital health information related to your condition? Please describe your experiences and any factors that help or hinder your use of such information.
2. What are your experiences of talking to other people about HIV? Have you told anyone about your HIV status, and if so, who? Can you tell me about their reactions?
3. Can you tell me about situations when you feel good? Can you tell me about situations when you not feel good?

(Probe: explore examples of such situations at home, school, and healthcare setting)

1. What is your experience about other people’s perception of people living with HIV? Does this affect your ability to take medications, receive healthcare services that are important to your health, and form friendships with others? How do you manage your feelings in this regard?
2. How would you describe your support from home, school, friends, health facility etc.?

(Probe: explore additional support needs and use of digital technology to provide needed support)

1. How does having HIV affect your inner strength (i.e., the power you have inside to handle challenges), how you feel about yourself, ability to make and maintain friendships, and your development and future goals?

***Socio-demographic and clinical characteristics of caregivers***

***Date of interview*** ________________***Name of the hospital _____________________________***

| **#** | **Item** | **Response** |
| --- | --- | --- |
|  | **Background Characteristics** |  |
|  | Respondent gender | \| ___ \| |
|  | How old were you on your last birthday? **(Record age in complete years)** | \| ___ \| years |
|  | Educational status? | *\|_____________________ \|* |
|  | What is your current marital status? | 1. Never married. 2. Currently married. 3. Divorced/widowed. |
|  | What is your relationship with the adolescent?  a) Mother  b) Father  c) Grand parent  d) Other, please state | \|______________________\| |
|  | **Clinical information** |  |
|  | Adolescent interview code (To be used to link with caregiver interview) |  |
|  | *May I ask if you know your HIV status?* | 1. *Yes* 2. *No* |
|  | *What is your current HIV test result?*  *(Complete as either* ***positive*** *or* ***negative****)* | \|__________________\|  Skip to the KII if “Negative” |

***KII Guides: Caregivers’ perspectives on anti-HIV stigma health support needs***

1. Can you describe your child’s involvement in psychosocial support groups at the hospital? What is your experience when discussing the topic of support with your child?
2. What is your experience of psychosocial support needs of adolescents living with HIV? Can you describe some of the challenges your child face in attending this support group?
3. What has been your experience in accessing health information about HIV? Please share your insights and any experiences you have had utilizing digital platforms as information source?

(Probe: whether the caregiver discusses with the child regarding the health information received related to HIV; suggestion on specific HIV-related health information and support needs of adolescents living with HIV)

1. What are your thoughts regarding the attitudes held by people towards individuals living with HIV in different contexts (for example, at community, home, school, and healthcare setting) and how do these attitudes affect the care and support of adolescents living with HIV?
2. Can you tell me about the general support available to adolescents living with HIV in different contexts such as home, school, friends, or at health facilities?

(Probe: explore additional support needs and use of digital technology to provide needed support)

1. In your experience, could you elaborate on how being HIV-positive affects adolescents in terms of their inner strength, self-perception, ability to form and maintain friendships, as well as their personal development and aspirations for the future?

**Socio-demographic characteristics of healthcare providers**

***Date of interview ________________Name of the hospital _____________________________***

| **#** | **Item** | **Response** |
| --- | --- | --- |
|  | **Background Characteristics** |  |
|  | Respondent gender | \| ___ \| |
|  | How old were you at your last birthday?  **Record age in complete years** | \| ___ \| |
|  | What is your current marital status? (Type answers in the space provided) | 1. Never married. 2. Currently married. 3. Divorced/widowed. |
|  | Educational status? | \|_____________________________\| |
|  | What is your profession? | \|_____________________________\| |
|  | What is your responsibility in this health facility? | \|_____________________________\| |
|  | Years of experience working with children and adolescents living with HIV? | \| ___ \| |

**KII Guides: Healthcare providers’ perspectives on anti-HIV stigma support needs**

1. Can you describe your experience of working with adolescents living with HIV?
2. What is your experience of psychosocial support to adolescents living with HIV?

(Probe: explore on barriers and facilitators and areas of improvement of psychosocial support to adolescents living with HIV).

1. What is your opinion on use of digital technology (e.g., mobile phone) to provide HIV related health information to adolescents living with HIV?

(Probe: explore on barriers and facilitators; type of information that can be delivered; approach (modality) to deliver such information).

1. What is your experience about other people’s attitudes towards adolescents living with HIV in different settings like home, school, and healthcare settings?
2. Can you tell me about the general support available to adolescents living with HIV in different contexts such as home, school, friends, or at health facilities?

(Probe: explore additional support needs and use of digital technology to provide needed support)

1. In your experience, could you elaborate on how being HIV-positive affects adolescents in terms of their inner strength, self-perception, ability to form and maintain friendships, as well as their personal development and aspirations for the future?
